# Peer Review of “Dental Tissue Density in Healthy Children Based on Radiological Data: Retrospective Analysis”

**DOI:** 10.2196/60329

**Published:** 2024-06-20

**Authors:** Shanmukha Gorthy

**Affiliations:** 1 Drs Sudha & Nageswara Rao Siddhartha Institute of Dental Sciences, Chinoutpalli, India

**Keywords:** density, teeth, tooth, dental, dentist, dentists, dentistry, oral, tissue, enamel, dentin, Hounsfield, pathology, pathological, radiology, radiological, image, images, imaging, teeth density, Hounsfield unit, diagnostic imaging

*This is the peer-review report for* “*Dental Tissue Density in Healthy Children Based on Radiological Data: Retrospective Analysis.”*

## Round 1 Review

### General Comments

This paper [1] has a very good topic selected by the authors. Based on the study of Hounsfield units in cone-beam computed tomography (CBCT), we can evaluate conditions that are abnormal in patients.

### Specific Comments

There is not much to comment on, but try to include good pictures of the CBCT.

#### Major Comments

1. The authors did not mention the ideal values of the Hounsfield units for enamel and dentin.

2. The authors did not properly explained why there were no gender differences.

3. The authors did not explain why there were the fewest density values for second primary molars and the most density values for maxillary central incisors.

## Abbreviations

CBCT: cone-beam computed tomography
